# A Comprehensive Analysis of the Interrelationship Between Craniofacial Variables in Cephalometric Analysis and Obstructive Sleep Apnea (OSA)

**DOI:** 10.3390/jcm14061963

**Published:** 2025-03-14

**Authors:** Olja Tanellari, Adela Alushi, Sara Ghanim, Carina Balcos, Daniel Petru Cioloca, Irina Nicoleta Zetu

**Affiliations:** 1 Department of Orthodontics, Faculty of Dental Medicine, “Grigore T. Popa” University of Medicine and Pharmacy, 700115 Iasi, Romania; oli_koca@yahoo.com (O.T.); nicoletazetu@gmail.com (I.N.Z.); 2 Department of Orthodontics, Faculty of Dental Medicine, University of Medicine, 1005 Tirana, Albania; adela.alushi@ual.edu.al; 3 Operator for Healthcare Services (OSHKSH), 1000 Tirana, Albania; ghanimsara@yahoo.com; 4 Department of Surgery, Faculty of Dental Medicine, “Grigore T. Popa” University of Medicine and Pharmacy, 700115 Iasi, Romania; daniel.cioloca@umfiasi.ro

**Keywords:** obstructive sleep apnea, cephalometry, airway obstruction, craniofacial abnormalities

## Abstract

**Background/Objectives**: Obstructive sleep apnea syndrome (OSAS) is a global condition usually associated with poor health. While common, it appears underdiagnosed due to repeated episodes of upper airway obstruction during the sleep cycle. It is accompanied by other health risks like cardiovascular issues and conditions. Identifying craniofacial characteristics linked to OSAS may enhance diagnostic precision and treatment planning. The aim of our study was to examine the relationship between cephalometric variables and OSAS and determine whether craniofacial features influence the syndrome’s development and severity. **Methods**: Thirty participants were split into two groups: 15 diagnosed with OSAS and 15 controls. Cephalometric evaluations were performed using standardized lateral imaging, with craniofacial, dental, and hyoid bone parameters assessed. Statistical analysis compared these variables between groups to identify significant differences. **Results**: OSAS patients exhibited significantly shorter maxillary and mandibular lengths, increased anterior facial height, and reduced posterior facial height compared to controls. Dental analysis revealed reduced interincisal angles and lower mandibular incisor positions in the OSAS group. The hyoid bone was positioned lower and more posteriorly in OSAS patients, with significant differences in its distance to the C3 vertebra and mandibular plane. Although the soft palate dimensions were larger in OSAS patients, the differences were not statistically significant. **Conclusions**: OSAS is associated with distinct craniofacial features, including shorter maxillary and mandibular lengths, altered facial height proportions, and lower hyoid bone positioning. These findings suggest that craniofacial morphology plays a significant role in OSAS pathophysiology. Future studies hinting at three-dimensional imaging could provide deeper insights into these associations.

## 1. Introduction

Obstructive sleep apnea syndrome (OSAS) is a common variation of a sleep disorder that has to do with breathing; however, it is poorly diagnosed and treated. Its impact on personal as well as public health is of paramount importance. It features repeated periods of sleep with obstructive (hypopnea) or complete (apnea) airflow cessation, which is a result of upper airway obstruction during sleep, subsequently triggering hypoxia and hypercapnia. In some cases, there is a real challenge to restore normal respiration [1,2,3].

OSAS severity is measured using the Apnea-Hypopnea Index (AHI), with over five events per hour deemed abnormal. The etiology of OSAS involves genetic, anatomical, and environmental factors, including age, gender, obesity, and dentofacial anomalies affecting airway dynamics. The condition is linked to excessive daytime sleepiness, cognitive impairments, metabolic issues, and cardiovascular risks, imposing substantial health and economic burdens. Despite its prevalence, OSAS remains underdiagnosed, emphasizing the need for improved awareness and diagnostic strategies [4].

While polysomnography (PSG) is the gold standard diagnostic, providing comprehensive data on sleep and respiratory patterns, it does not localize airway obstructions. Advanced imaging techniques like computer tomography (CT), magnetic resonance imaging (MRI), cephalograms, and naso-laryngoscopy are thus required for precise obstruction localization, bringing additional benefit in treatment planning and prognosis [5]. Despite its role as a valuable gold standard diagnostic technique for obstructive sleep apnea, PSG is rather constrained by its expensive, time-consuming process and the requirement of performing the test in a potentially strange environment for the patient.

Therefore, for the early identification and severity evaluation of obstructive sleep apnea (OSA), quicker, non-invasive, less expensive alternative approaches have been suggested. These include sleep questionnaires, which have demonstrated high specificity and sensitivity in diagnosing OSA, particularly in regions lacking specialized sleep centers. Screening tools, like the Epworth Sleepiness Scale (ESS) and STOP-Bang questionnaire, facilitate early OSAS detection in non-specialized settings. The ESS quantifies daytime sleepiness, a key OSAS symptom, while the STOP-Bang questionnaire assesses risk factors such as snoring, fatigue, apneas, hypertension, body mass index (BMI), age, neck circumference, and gender. These tools, particularly STOP-Bang, exhibit high sensitivity and specificity, enabling timely identification and intervention [6]. Additionally, profile teleradiography, a routine diagnostic tool in orthodontics, can reveal morphological changes that are directly associated with sleep apnea.

Cephalometric analysis is also valuable in identifying craniofacial traits associated with OSAS, such as mandibular retrognathia, micrognathia, increased ANB angle, lowered hyoid bone, and reduced pharyngeal space. However, despite these available diagnostic tools, many cases remain undetected, highlighting the need for effective screening [7].

The goal of this study was to investigate the relationship between cephalometric factors and the obstructive sleep apnea syndrome, in order to determine if craniofacial traits can impact OSA.

## 2. Materials and Methods

### 2.1. Patient Selection

The study sample included 30 participants, divided into two groups: the OSA group (*n* = 15) and the control group (*n* = 15).

Data were retrospectively collected from patient records at two private hospitals, where individuals had been diagnosed with sleep apnea syndrome following polysomnographic assessments. The OSA group included patients diagnosed with obstructive sleep apnea, while the control group comprised individuals who were suspected of having sleep apnea but were subsequently determined to be healthy after undergoing polysomnography. Inclusion and exclusion criteria are listed in Table 1.

### 2.2. Study Design

The retrospective study was conducted in accordance with the Declaration of Helsinki and approved by the Institutional Ethics Committee of University of Medicine and Pharmacy “Grigore T. Popa” from Iasi, Romania (Nr. 204/03 July 2022). This research adhered to the principles outlined in the Declaration of Helsinki. Patients were provided with both verbal and detailed written explanations of the study and provided consent to participate in the study. Information was gathered from observation charts for a range of symptoms associated with obstructive sleep apnea, over the course of a year.

The AHI was used to assess the severity of obstructive sleep apnea (OSA), while oxygen desaturation levels were used as a biomarker for the degree of impairment associated with OSA [8]. The AHI, which is reported as the number of events per hour, measures the frequency of apnea and hypopnea episodes per hour of sleep. Based on AHI, OSA severity is classified into four categories: mild (AHI ≥ 5 but <15 events/hour), moderate (AHI ≥ 15 but <30 events/hour), severe (AHI ≥ 30 events/hour), and none/minimal (AHI < 5 events/hour). Every participant’s BMI was within normal limits.

To ensure procedural uniformity, a single technician conducted a standardized lateral cephalometric examination. Measurements were carried out during the expiratory phase of breathing when the head was in a neutral position with the teeth in maximal intercuspation. Since the results were evaluated by a single examiner, the risk of bias was minimized by using standardized protocols and manually tracing craniometric landmarks to ensure measurement consistency. The evaluator followed strict instructions, and intra-examiner reliability was verified by repeating certain measurements and analyzing correlations. Carestream Dental’s (Carestream Dental LLC, Atlanta, GA, USA) proprietary software, CS 3D Imaging and CS Airway Imaging version 7.0.23, were used to evaluate the cephalometric data [9].

The detailed craniometric parameters assessed are outlined below (Table 2).

After analyzing the files, 15 patients with proven OSA were selected after polysomnographic testing and subjected to cephalometric study. The other 15 patients suspected of having apnea, who were deemed healthy after polysomnography, comprised the control group.

In terms of demographic data, patients in the OSA group were 51.5 ± 11.01 years old on average, whereas those in the control group were 48.6 ± 9.74 years old. In the OSA group, the female-to-male ratio was 8:7, whereas in the control group, it was 9:5 (Table 3).

### 2.3. Statistical Analysis

All cephalograms were manually traced by a single operator, to ensure consistency. The data are reported as mean values accompanied by their standard deviations. The collected data were analyzed with SPSS 26.0 (IBM SPSS Statistics, NY, USA), where all statistical analyses were conducted. Cephalometric characteristics were compared between patients with obstructive sleep apnea (OSA) and control volunteers, using independent *t*-tests. Statistical significance was defined as a *p*-value of less than 0.05.

## 3. Results

There were small differences in SNA, SNB, and ANB measures across groups. In particular, the OSA group had lower SNB values than the control group, whereas the SNA and ANB values were greater. However, as shown by the corresponding *p*-values of 0.356, 0.275, and 0.112, these differences fell short of statistical support (Table 4).

With mean values of 86.20 ± 6.26 and 89.33 ± 6.77, respectively, the analysis pointed out that the OSA group’s maxilla dimensions were substantially lower than those of the control group; therefore, the difference was statistically significant (*p* = 0.022). Mandibular size also recorded significant differences, with the OSA group revealing a lower mean value (97.5 ± 4.5) compared to control group (108.6 ± 5.8), with *p* < 0.05 (*p* = 0.009). Additionally, there were variations in midface height (N-SNA), with the OSA group recording a higher difference (*p* < 0.05). Additionally, the Go-Me/S-N measures indicated that face hyperdivergence was present in OSA patients (Table 5).

The results of the cephalometric analysis showed that the OSA and control groups differed significantly in vertical facial height, measured both anteriorly and posteriorly. The OSA group presented a greater anterior facial height (138.83 ± 9.06) than the control group (134.64 ± 11.06), with a statistically significant difference (*p* = 0.004), and a lower posterior facial height (87.33 ± 8.82) than the control group (90.25 ± 7.17), with the difference being more statistically significant for the former (*p* = 0.004) (Table 6).

There were statistically significant differences between the two groups, according to the dental study. With mean values of 125.89 ± 8.16° and 132.85 ± 9.88°, respectively, the OSA group’s interincisive angle was substantially lower than that of the control group, resulting in a statistically significant difference (*p* = 0.000). Additionally, there was a statistically significant difference (*p* = 0.002) between the OSA group and the control group in terms of the mandibular incisor position (L1-NB), with mean values of 2.89 ± 4.67 mm and 5.36 ± 3.46 mm, respectively. On the other hand, there was no discernible difference between the two groups’ maxillary incisor positions (U1-NA) (Table 7).

The hyoid bone is a key landmark in assessing craniofacial changes in OSA. In our study, significantly greater distances from the hyoid bone to fixed anatomical reference points in the OSA group were identified, indicating a lower hyoid position. Notable differences were observed in the distances to the C3 vertebra (H-C3: 36.4 ± 1.9 mm vs. 32.3 ± 2.2 mm, *p* = 0.032) and the mandibular plane (H-MnP: 22.8 ± 5.2 mm vs. 19.5 ± 5 mm, *p* = 0.017) (Table 8).

In the OSA group, a larger size of the soft palate was noted, compared to the control group, with increased length and thickness observed (PNS-UT: 42.71 ± 5.14 vs. 41.66 ± 5.47 and Max U: 9.84 ± 2.02 vs. 9.24 ± 1.95, respectively). However, the recorded differences did not reach statistical significance (Table 9).

## 4. Discussion

Obstructive sleep apnea (OSA) is characterized by repeated complete or partial obstructions of the upper airway, leading to oxygen desaturation and sleep arousals. These disturbances contribute to common symptoms such as excessive daytime sleepiness, fatigue, and impaired concentration [2]. Upper airway constriction is a significant component of the complex interaction between anatomical and non-anatomical factors that make up the pathophysiology of OSA. Although the exact differences across races are still unclear, the etiology of OSA may vary [10,11]. Constricted skeletal frameworks and hypertrophied soft tissues may result in the constriction of the upper airway. The anatomical balance of the upper airway, defined by the ratio of tongue size to maxillomandibular enclosure size, plays a pivotal role in determining airway shape and collapsibility [12,13].

### 4.1. Facial Skeletal Discrepancies in OSA Patients

Cephalometry and 3D-CT imaging can evaluate craniofacial skeletal anomalies and oropharyngeal soft tissue properties. Despite its restricted two-dimensional perspective, cephalometry is a straightforward and readily available assessment technique that entails decreased radiation exposure, compared to computed tomography [14]. A meta-analysis of cephalometric studies conducted by Miles et al. [15] identified key variables potentially linked to the onset and severity of OSA: anterior skull base-maxillary angle (ASNA), anterior skull base-mandibular angle (ASB-M), posterior airway space (PAS), soft palate length (P-S), and hyoid bone-to-mandibular plane distance (H-P). The authors showed a reduction in mandibular and maxillary length. In people with OSA, Neelapu et al. [16] found a substantial correlation between AHI and craniofacial measures, such as the Tragion-Ramus-Stomion angle, face width ratio, and cervicomental contour ratio.

The maxilla exhibits a reduced length, accompanied by a narrower and more conical maxillary arch. Mandibular retrusion is also linked to OSA, with three-dimensional imaging confirming a reduced mandibular closure area in affected individuals. The spatial relationship between the maxilla and mandible is significant in the etiology of OSA. The prevalent cephalometric characteristic to be documented is the inferior displacement of the hyoid bone, or the augmented distance between the mandibular plane and the hyoid [17].

Studies indicate that, while OSA severity is similar, diagnosed people are more likely to be overweight, as indicated by higher BMI and neck circumference. On the other hand, Asian adults with OSA exhibit more noticeable bony constrictions, which are indicated by a smaller retrognathic mandible and maxilla. According to recent studies exhibiting skeletal differences between OSA patients and control groups in the sagittal and vertical planes, these anatomical differences place the entire facial complex closer to the cervical spine, with subsequent reduction of the amount of available airway space in both sleep-disordered breathing cohorts [18,19].

Liu et al. [20] described the craniofacial and cephalometric characteristics seen in OSA patients and how they affect the use of mandibular advancement devices to treat OSA. The authors proposed two categories of craniofacial and soft tissue morphologies in patients with OSA: those who present with maxillary retrusion, an enlarged oropharynx, a larger soft palate, and over-erupted maxillary molars; and those who exhibit maxillary prognathism along with a diminutive oropharynx, under-erupted maxillary molars, overbite, and a small soft palate.

There is substantial evidence of modified dimensions and placement of the maxilla and mandible in people with OSA. The present investigation indicated a reduction in the SNB angle, reduced mandibular size, and clockwise rotation of the mandible in participants with OSA. A reduction in jaw length was noted; however, its sagittal position remained normal. A reduced cranial base, indicating a diminutive jaw in OSA sufferers, correlates with a standard SNA score [17,21,22].

Patients with OSA exhibit a pronounced inclination towards increased anterior facial heights [23,24]. Meta-analyses on this subject have authenticated a substantial increase in overall anterior facial height [25,26] and posterior facial height [27,28,29], while upper anterior facial height exhibited a nonsignificant increase. An important discovery of the study was the elevation in anterior facial height at the midface level, which contributes to the overall increase in total anterior facial height. No substantial alterations were noted in posterior heights.

### 4.2. Hyoid Bone Position and Airway Patency

In patients with obstructive sleep apnea, the hyoid bone was situated at a lower position. This result aligned with prior research [30,31]. The hyoid bone’s position functions as a central anchor for the tongue muscles and dictates their placement. A lowered hyoid bone may indicate increased pressure from excess pharyngeal tissue on the craniofacial structure.

Conversely, the hyoid’s position may not serve as a predisposing factor, but rather as a physiological adaptation to preserve airway patency; for instance, it may facilitate the displacement of the tongue’s dorsum and soft palate away from the posterior pharyngeal wall, to mitigate the obstructive condition [32,33]. Paoli et al. [34] claimed that prolonged nocturnal pressure can lead to the elongation of the hyoid ligaments. Tsai et al. [35] asserted that a hyoid bone composed of hard tissue, readily discernible on radiographs, may serve as a superior prognostic marker for distinguishing between OSA and control groups, in contrast to the soft palate and dorsum of the tongue, which can occasionally appear ambiguous on standard lateral radiographs. Silva et al. [36] and Yucel et al. [37] concur with this assertion, identifying MP-H as a dependable metric for evaluating OSA. A diminished hyoid position results in increased tongue mass accumulation in the hypopharyngeal region, potentially serving as an unfavorable prognostic factor for the effective application of mandibular advancement devices [38]. Some authors reported that the hyoid bone is significant for preserving the proportions of the upper airway [39].

A reduced hyoid position, accompanied by a lowered tongue posture, may elevate mandibular strain due to the increased energy necessary for tongue elevation; this, consequently, may exacerbate apnea, leading to an open-mouth posture during sleep [40].

### 4.3. Soft Palate and Airway Dimensions

The present study found no significant differences in soft palate length between the OSA and control groups, which was consistent with the findings of previous studies [15]. According to Ivanhoe et al. [41], structural differences in the craniofacial structure that supports the airway may be the cause of the smaller upper airway dimensions in OSA patients, as compared to healthy individuals. Because the posterior pharyngeal wall and soft palate enclose the root of the tongue, airway narrowing often occurs when patients recline supine [26]. One common cause of snoring and OSA is an expanded soft palate or an excess of soft palate tissue [24].

In some studies, the soft palate length was significantly shorter in patients with obstructive sleep apnea than in the control group. Our study found no significant difference in soft palate thickness between the OSA group and the control group, but Battagel et al. [38] found a significant increase in soft palate thickness among OSA patients. The sizes of the tongue and soft palate are essential for the physiological maintenance of the upper airway, and hypertrophy of the tongue and soft palate causes lower upper airway dimensions in those with OSA.

The study presents important elements, including the use of a standardized methodology, measurements conducted by a single operator to reduce variability, and strict participant selection criteria. The statistical analysis strengthened the validity of the results, while the comparison of cephalometric variables between patients with OSA and the control group provided valuable insights into craniofacial correlations. However, the study also has limitations, such as a small sample size (30 participants), which may restrict the generalizability of the results. Additionally, the use of two-dimensional imaging does not fully capture the complexity of the airway, and future research should incorporate three-dimensional techniques (CBCT, MRI). A potential selection bias exists, as participants were recruited from private hospitals, and other factors, such as genetic predisposition and lifestyle, were not analyzed in detail.

This study’s cross-sectional design and reliance on cephalometric analysis may limit the universality of results. Future research testing three-dimensional imaging could provide more comprehensive insights into craniofacial and airway morphology in OSA patients.

To validate the findings regarding the use of three-dimensional imaging in analyzing craniofacial morphology and airway structures in patients with obstructive sleep apnea, longitudinal studies are needed to track structural changes over time, validation research to compare different 3D imaging techniques and correlate them with clinical data, clinical correlation studies to examine the relationship between morphological parameters and disease severity, comparative studies across populations to identify relevant differences, interventional research to assess the impact of treatments on airway morphology, as well as the integration of artificial intelligence and machine learning algorithms to optimize the analysis and interpretation of imaging data.

## 5. Conclusions

This study identifies significant craniofacial differences between OSA patients and healthy controls. The SNB angle was lower in the OSA group, while the SNA and ANB angles were higher. Mandibular–cranial relationships differed between groups, but these differences were not statistically significant. Maxillary and mandibular lengths were significantly shorter in OSA patients. Anterior facial height was increased at the mid-facial level, contributing to a greater total anterior facial height, while posterior facial height was significantly lower in OSA patients.

Dental examination revealed that the mandibular incisor location and interincisal angle varied significantly, with the OSA group displaying smaller angles. In OSA patients, the hyoid bone was positioned posteriorly and lower, with notable variations in the distances between the hyoid and the mandibular plane and C3 vertebra. In OSA patients, the soft palate was thicker and longer, although these variations were not statistically significant.

## Figures and Tables

**Table 1:** Inclusion and exclusion criteria.

Inclusion Criteria	Exclusion Criteria
- Subjects aged between 18 and 65 years - Patients diagnosed with obstructive sleep apnea	- Subjects aged under 18 or over 65 years, - Subjects who did not consent, - Patients with congenital anomalies (e.g., cleft palate, Pierre Robin syndrome), genetic syndromes, or obstructive sleep apnea with a history of orthodontic treatment. - Subjects with COPD, neurological or psychiatric conditions worsened by respiratory infections, with prior orthognathic surgery, or respiratory procedures

**Table 2:** The craniometric parameters used for the assessment of the cephalometric analysis.

Measurements	Variable Description
Maxilla and Mandible	
ANB	Indicate the skeletal relationship between the maxilla and mandible–it is a normal skeletal class I, II, or III.
SNA	Indicate whether the maxilla is normal, prognathic, or retrognathic in relation to the cranial base.
SNB	Indicate whether the mandible is normal, prognathic, or retrognathic in relation to the cranial base.
WITS (AO-BO)	Skeletal class
SNA-SNP	Maxillary length
S-N	Cranial base
Go-Me/S-N	Angle formed between the mandibular plane and the cranial base (facial divergence)
N-SNA (mm)	Middle anterior facial height
SNA-Me	ANS-Me (lower anterior facial height): the distance from ANS to Me.
Anterior facial length (Na-Me) (mm)	Distance between Nasion and Menton
Posterior facial length (S-Go) (mm)	Distance between Sella and Gonion
P-A facial length ratio (S-Go/N-Me) (%)	Ratio between S-Go and N-Me, expressed as a percentage.
Dental assessment	
U1-L1 (grade)	Interincisal angle
U1-Na (mm)	Distance between the most prominent upper incisor and the line connecting points N and A
L1-NB (mm)	Distance between the most prominent lower incisor and the line connecting points N and B
U1-NA (grade)	Angle formed between the most prominent upper incisor and the line connecting points N and A
L1-NB (grade)	Angle formed between the most prominent lower incisor and the line connecting points N and B
Uvula assessment	
PNS-UT	Uvula length (distance from PNS to UT)
Max U	Maximum uvula thickness (maximum thickness of the uvula perpendicular to its length)
Hyoid bone assessment	
H-A	Distance from H to A
H-B	Distance from H to B
H-MnP	Distance from H to the mandibular plane (Go-Me)
H-C3	Distance from H to C3

**Table 3:** Comparison of age distribution, female-to-male ratio, and AHI between the OSA group and control group.

	OSA Group	Control Group
Age	51.5 ± 11.01 years	48.6 ± 9.74 years
Gender female/male	8/7	9/5
AHI		
None/Minimal	2	15
Mild	6	
Moderate	7	
Severe	0	

**Table 4:** A comparison of the SNA, SNB, and ANB angles between the OSA group and control group.

Parameters	OSA Group	Range	Control Group	Interval	*p*-Value
ANB	4.53 ± 1.8	−3.64–4.97	3.49 ± 2.7	−1.4–6.7	0.356
SNA	82.93 ± 3.2	71.40–88.60	83.29 ± 3.0	77.40–89.00	0.275
SNB	78.4 ± 3.4	67.40–90.00	79.8 ± 2.9	70.50–81.5	0.112

**Table 5:** Assessment of maxilla and mandible dimensions, midface height, and facial divergence in individuals with OSA versus control group.

Parameters	OSA Group	Range	Control Group	Interval	*p*-Value
ANS-PNS	86.20 ± 6.26	56.90–93.50	89.33 ± 6.77	67.10–98.10	0.022
Mandibular dimension	97.5 ± 4.5	84.3–114.6	108.6 ± 5.8	94.7–113.5	0.009
Go-Me/S-N (facial divergence)	38.66 ± 7.36	21.60–50.40	32.11 ± 5.27	25.20–46.00	0.079
N-SNA	93.32 ± 5.91	83.4–106.9	88.39 ± 5.35	79.6–97.6	0.013

**Table 6:** Vertical facial height comparison in the OSA group and the control group.

	OSA Group	Range	Control Group	Interval	*p*-Value
Na-Me (mm)	138.83 ± 9.06	123.00–157.30	134.64 ± 11.06	119.9–159.2	0.004
S-Go (mm)	87.33 ± 8.82	69.10–97.30	90.25 ± 7.17	78.70–104.00	0.028
S-Go/N-Me (%)	65.03 ± 3.32	57.70–71.20	64.98 ± 5.70	51.40–74.10	0.144

**Table 7:** Dental evaluations in the OSA group compared to control group.

	OSA Group	Range	Control Group	Interval	*p*-Value
(U1-L1) (°)	125.89 ± 8.16	112.30–141.60	132.85 ± 9.88	117.60–150.10	0.000
U1-NA (mm)	4.68 ± 3.49	−2.80–8.50	3.18 ± 3.29	−2.00–10.30	0.254
L1-NB (mm)	2.89 ± 4.67	−6.10–9.60	5.36 ± 3.46	0.30–12.90	0.002
U1-NA (°)	23.03 ± 6.16	11.40–31.50	17.10 ± 7.35	6.00–29.30	0.001
L1-NB (°)	25.18 ± 7.16	14.80–38.00	24.98 ± 7.06	15.10–39.60	0.104

**Table 8:** Hyoid bone evaluations in the OSA group compared to control group.

	OSA Group	Control Group	*p*-Value
H-A	41.3 ± 3.4	40.6 ± 2.7	0.247
H-B	16.85 ± 1.14	15.3 ± 2.1	0.189
H-MnP	22.8 ± 5.2	19.5 ± 5	0.017
H-C3	36.4 ± 1.9	32.3 ± 2.2	0.032

**Table 9:** Soft palate assessment.

	OSA Group	Range	Control Group	Interval	*p*-Value
PNS-UT	42.71 ± 5.14	34.40–52.00	41.66 ± 5.47	34.50–56.80	0.168
Max U	9.84 ± 2.02	6.70–13.30	9.24 ± 1.95	6.90–14.70	0.223

## Data Availability

Data are contained within the article.
